# Supplementary material for: Uncovering Functional Contributions of PMAT (Slc29a4) to Monoamine Clearance Using Pharmacobehavioral Tools
Source: Cells. 2022 Jun 9;11(12):1874. doi: 10.3390/cells11121874 (PMC9220966; doi:10.3390/cells11121874)
Supplement: Supplementary file 1 [file cells-11-01874-s001.zip › PMAT PAD Supplemental Resub R1.pdf]

Supplemental Material for:

Uncovering Functional Contributions of PMAT (Slc29a4) to Monoamine Clearance Using Pharmacobehavioral Tools

Jasmin N. Beaver, Brady L. Weber, Matthew T. Ford, Anna E. Anello, Sarah K. Kassis and T. Lee Gilman *

Department of Psychological Sciences & Brain Health Research Institute, Kent State University, Kent, OH, 44242, USA

*Corresponding Author

T. Lee Gilman, Ph.D.

tgilman@kent.edu

209 Kent Hall Addition

600 Hilltop Dr.

Department of Psychological Sciences

Kent State University

Kent, OH 44242

At the request of the expert reviewers of this manuscript, we are including here an extended presentation of our post-tail suspension test (TST) locomotor testing. This was done in response to an inquiry regarding whether plasma membrane monoamine transporter (PMAT) might influence the pharmacokinetics of the antidepressant drugs used, escitalopram and bupropion. Though certainly not a substitute for blood nor tissue measurements of drug concentrations, assessment of locomotor activity in response to different doses of these drugs might indicate variability in pharmacokinetics as a consequence of constitutive PMAT deficiency.

To supplement the data in the primary manuscript (Figure 3) assessing locomotor activity mirroring the window of time at which TST testing occurs post-drug injection (i.e., 30-40 min), we present here the entire time course of post-TST locomotor testing (Supplemental Figure S1). For clarity, and to assess within each sex and drug treatment how locomotor activity was affected across genotypes, we performed two-way repeated measures ANOVAs (time × genotype) with Greenhouse-Geisser corrections for within-subjects analyses. Data are shown in Supplemental Figure S1, with the grey shaded area indicating those data used for Figure 3. Only in males at the higher doses of escitalopram was there a significant time × genotype interaction (plus a trend towards and interaction at the higher bupropion dose in males), suggesting the potential for a pharmacokinetic influence of PMAT deficiency on the behavioral response to escitalopram (for all statistics, see Supplemental Table S1). However, Tukey's post-hoc testing indicated that only for the 25–30 min bin was there a significant ($p = 0.0477$) increase in locomotor activity in male heterozygotes after 2 mg/kg escitalopram relative to male wildtypes.

Supplementary Table S1. Statistical analyses of post-tails suspension test locomotor testing.

	Females	Males
Saline		
Time × Genotype	F(22,242)=0.08258, p=0.6921	F(22,275)=0.7821, p=0.7473
Time	F(5.392,118.6)=4.177, **p=0.0012**	F(6.606,165.2)=1.915, p=0.0743
Genotype	F(2,22)=0.02758, p=0.9728	F(2,25)=0.9110, p=0.4151
1 mg/kg Escitalopram		
Time × Genotype	F(22,231)=0.5796, p=0.9348	F(22,275)=0.8540, p=0.6560
Time	F(5.419,113.8)=3.428, **p=0.0051**	F(5.830,145.8)=5.393, **p<0.0001**
Genotype	F(2,21)=1.379, p=0.2738	F(2,25)=8.642, **p=0.0014**
2 mg/kg Escitalopram		
Time × Genotype	F(22,231)=0.8147, p=0.7061	F(22,264)=2.022, **p=0.0052**
Time	F(5.939,124.7)=4.773, **p=0.0002**	N/A
Genotype	F(2,21)=0.09089, p=0.9135	N/A
4 mg/kg Bupropion		
Time × Genotype	F(22,231)=1.201, p=0.2478	F(22,297)=0.6459, p=0.8889
Time	F(5.317,111.7)=7.309, **p<0.0001**	F(5.269,142.3)=6.774, **p<0.0001**
Genotype	F(2,21)=0.02435, p=0.9760	F(2,27)=4.619, **p=0.0188**
8 mg/kg Bupropion		
Time × Genotype	F(22,242)=0.9994, p=0.4659	F(22,275)=1.519, p=0.0666
Time	F(5.375,118.2)=5.805, **p<0.0001**	F(3.628,90.71)=11.90, **p<0.0001**
Genotype	F(2,22)=3.748, **p=0.0398**	F(2,25)=1.772, p=0.1906

Statistics corresponding to data graphed in Supplementary Figure S1. Two-way repeated measures ANOVAs with Greenhouse-Geisser corrections for within-subjects analyses were performed within sex (columns) and treatment (rows headers) to assess if significant ($p<0.05$; **bolded** p values in table) interactions between time × genotype were present. Significant interactions of time × genotype suggest a potential influence of PMAT deficiency on pharmacokinetics of respective drug treatment.

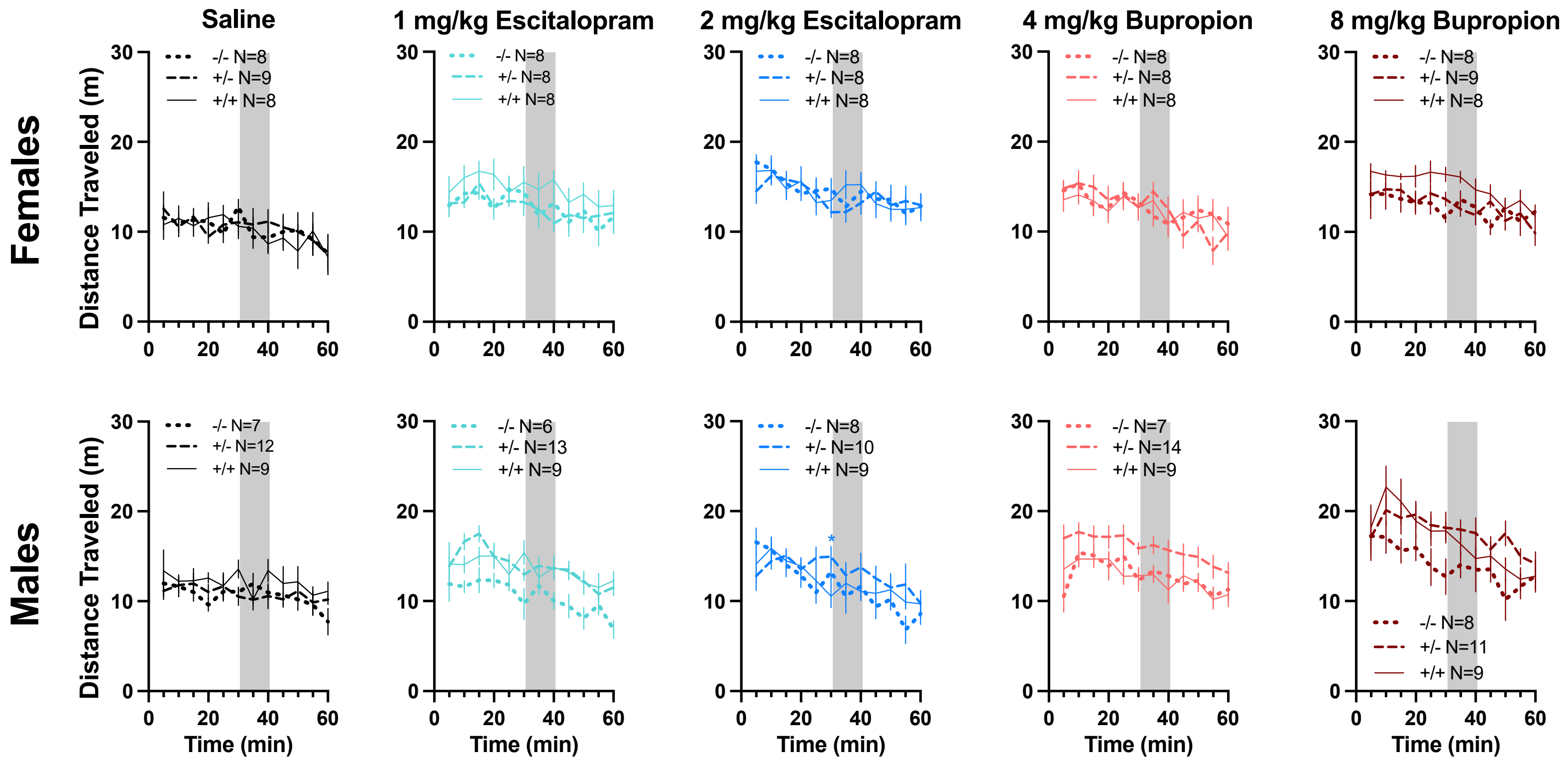

Supplemental Figure S1. Time course of locomotor activity in open field 8 days after tail suspension test.

Locomotor behavior in the open field across 5 min bins starting immediately after an injection of saline (10 mL/kg; black lines), escitalopram (light blue, 1 mg/kg; dark blue, 2 mg/kg), or bupropion (light red, 4 mg/kg; dark red, 8 mg/kg). Grey shaded area indicates time period that corresponds to when tail suspension test occurred relative to time of injection (i.e., 30-40 min later). Solid lines PMAT wildtype mice (+/+), dashed lines indicate PMAT heterozygote mice (+/−), and dotted lines indicate PMAT knockout mice (−/−). The top row is female data, and the bottom row is male data. Each graph was analyzed with a two-way repeated measures ANOVA (see Supplementary Table S1 for statistics). Data are shown as means ± SEM. * $p = 0.0477$ vs. wildtype males treated with 2 mg/kg escitalopram at same time point.
